# Genetic association between plasminogen activator inhibitor‐1 rs1799889 polymorphism and venous thromboembolism: Evidence from a comprehensive meta‐analysis

**DOI:** 10.1002/clc.23282

**Published:** 2019-11-08

**Authors:** Guangbin Huang, Pan Wang, Tao Li, Xuejun Deng

**Affiliations:** ^1^ Department of Trauma Surgery, Emergency Medical Center of Chongqing The Affiliated Central Hospital of Chongqing University Chongqing China; ^2^ Department of Cardiology Suining Central Hospital Suining China

**Keywords:** meta‐analysis, plasminogen activator inhibitor‐1 (*PAI‐1*), polymorphism, venous thromboembolism (VTE)

## Abstract

**Background:**

Association between plasminogen activator inhibitor‐1 (*PAI‐1*) rs1799889 polymorphism and venous thromboembolism (VTE) were explored by many previous studies, yet the findings of these studies were conflicting.

**Hypothesis:**

*PAI‐1* rs1799889 polymorphism may serve as a genetic marker of VTE. We aimed to better clarify the relationship between *PAI‐1* rs1799889 polymorphism and VTE in a larger combined population by performing a meta‐analysis.

**Methods:**

Literatures were searched in Pubmed, Embase, Web of Science, and China National Knowledge Infrastructure (CNKI). We used Review Manager to combine the results of individual studies.

**Results:**

Forty‐eight studies involving 14 806 participants were eligible for inclusion. Combined results revealed that *PAI‐1* rs1799889 polymorphism was significantly associated with VTE in Caucasians (dominant comparison: odds ratio [OR] 1.20, 95% confidence interval [CI] 1.09‐1.32; recessive comparison: OR 0.84, 95% CI 0.76‐0.94; allele comparison: OR 1.08, 95% CI 1.02‐1.15) and East Asians (dominant comparison: OR 1.60, 95% CI 1.17‐2.19; allele comparison: OR 1.53, 95% CI 1.21‐1.93). Further analyses obtained similar significant associations in these with deep vein thrombosis (DVT) and these with Factor V Leiden mutation.

**Conclusions:**

Our findings supported that *PAI‐1* rs1799889 polymorphism may serve as one of the predisposing factors of VTE in both Caucasians and East Asians, especially in these with DVT and these with Factor V Leiden mutation.

## 1 INTRODUCTION

Venous thromboembolism (VTE) is a common and severe thrombotic disorder. Its age‐ and sex‐adjusted incidence rate is estimated to be around 1.2‐1.4 per 1000 person‐years, and its 30‐day mortality rate could reach up to 10%.^2^, ^3^ Previous studies demonstrated that agedness, major surgical operations, cancer, gestation, and sequential oral contraceptives could increase susceptibility to VTE.^4^, ^5^ Nevertheless, many people who were exposed to these risk factors did not ultimately develop VTE, which suggested that genetic factors were also involved in its development.

Plasminogen activator inhibitor‐1 (PAI‐1) is a serine protease inhibitor, and it is involved in the regulation of fibrinolysis and thrombosis via inhibiting biological activities of tissue plasminogen activator (t‐PA) and urokinase plasminogen activator (u‐PA).^6^ Previous basic researches showed that blockage of PAI‐1 could lead to thrombus degradation, whereas activation of PAI‐1 could accelerate thrombus formation.^7^, ^8^ So if a gene polymorphism could alter the expression level or protein structure of PAI‐1, it is possible that this polymorphism may also affect individual susceptibility to thrombotic disorders like VTE.

In recent years, many investigations reported findings regarding potential associations between *PAI‐1* rs1799889 A/G polymorphism and VTE.^9^, ^10^, ^11^, ^12^ Nevertheless, these findings were somehow inconsistent. In this meta‐analysis, we aimed to better clarify the relationship between *PAI‐1* rs1799889 A/G polymorphism and VTE. We will also perform comprehensive analyses to investigate the effects of ethnic background, type of disease, and established risk factors of VTE (Factor V Leiden mutation, cancer status, and recent major surgery) on genetic association between *PAI‐1* rs1799889 A/G polymorphism and VTE.

## 2 MATERIALS AND METHODS

This meta‐analysis was written in accordance with PRISMA guideline.^13^ We also created an Open Science Framework (osf.io) account to make this meta‐analysis more publicly available.

### 2.1 Literature search and inclusion criteria

We searched PubMed, Web of Science, Embase, and CNKI using the following key words: “plasminogen activator inhibitor‐1,” “PAI‐1,” “plasminogen activator inhibitor 1,” “PAI1,” “serpin family E member 1,” “SERPINE1,” “polymorphism,” “variant,” “variation,” “mutation,” “SNP,” “venous thromboembolism,” “VTE,” “deep vein thrombosis,” “DVT,” “pulmonary embolism,” and “PE.” The latest literature searching update was conducted in June 2019.

To be included in this meta‐analysis, some criteria must be met: (a) About *PAI‐1* rs1799889 A/G polymorphism and VTE in human beings; (b) providing distributions of genotypes or alleles in cases and controls; (c) Full text in English or native language of the authors (Chinese) is retrievable. Studies were deemed to be ineligible for inclusion if: (a) Not about *PAI‐1* rs1799889 A/G polymorphism and VTE; (b) studies that were not carried out in humans; (c) case reports or case series; (d) reviews and comments. If we found repeated publications during literature searching, only the most comprehensive study was included for analyses.

### 2.2 Data extraction and quality assessment

Following information was extracted by two authors: the last name of the first author and publication year, country of the principal investigator and ethnicity of study participants, type of disease, total sample size of each study, and the distribution of *PAI‐1* rs1799889 A/G polymorphism in cases and controls. We also calculated the probability value (*P* value) of Hardy‐Weinberg equilibrium.

The authors used Newcastle‐Ottawa scale (NOS) to assess the quality of eligible studies.^14^ The score range of NOS is between zero and nine, when a study got a score of seven or more, we considered that the methodology of this study is good.

Two authors extracted data and assessed quality of eligible publications. The authors wrote to the leadings authors for additional information if essential information was found to be incomplete.

### 2.3 Statistical analyses

Review Manager was used to combine the results of eligible studies. *Z* test was employed to assess whether *PAI‐1* rs1799889 A/G polymorphism was significantly associated with VTE, with the statistical significance *P* level set at .05. We used *I* ^2^ statistics to assess between‐study heterogeneities. We used Random‐effect models (DerSimonian‐Laird method) to combine the results if *I* ^2^ is larger than 50%. Otherwise, fixed‐effect models (Mantel‐Haenszel method) were used to combine the results. We also conducted subgroup analyses by ethnicity of participants, type of disease and whether the study subjects had established risks of VTE. We examined the stability of combined results by deleting one study each time and combining the results of the remaining studies. We used funnel plots to estimate whether our combined results may be influenced by publication biases.

This article does not contain any studies with human participants or animals performed by any of the authors, thus ethical approval is not required.

## 3 RESULTS

### 3.1 Characteristics of included studies

**Figure 1:**
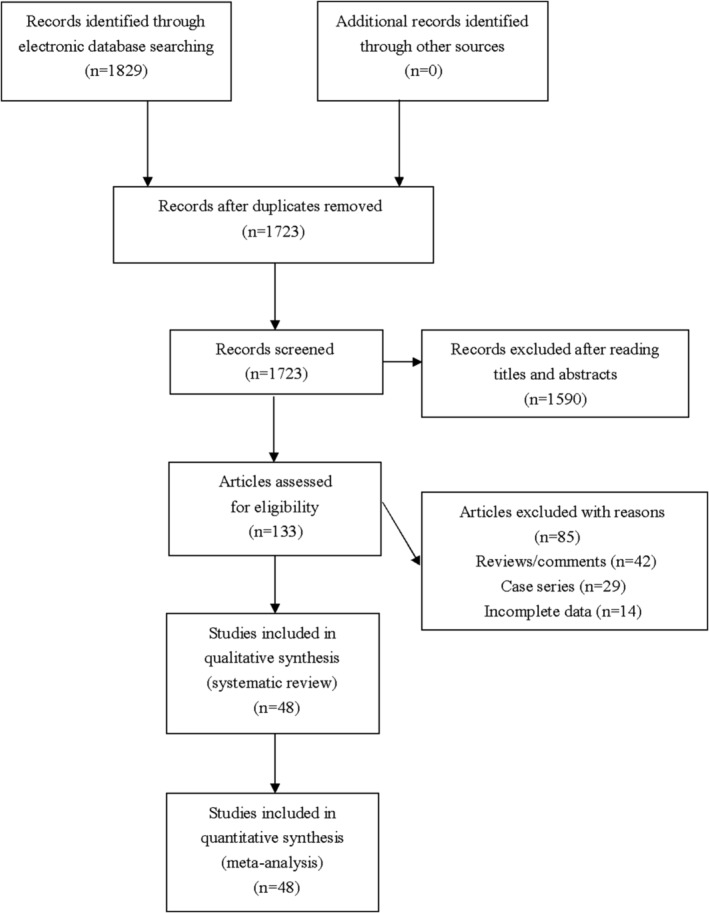
Flowchart of study selection for the present study

One thousand eight hundred and twenty‐nine studies were identified by our comprehensive literature searching. One hundred and thirty‐three studies were retrieved for eligibility assessment after exclusion of irrelevant and duplicate articles. Another eighty‐five articles were further excluded by us because these articles did not meet the inclusion criteria that were set forth for this meta‐analysis. Totally forth‐eight studies containing 5731 cases and 9075 controls were ultimately included in this meta‐analysis (see Figure 1). Table 1 presented essential data extracted from included studies.

**Table 1:** The characteristics of included studies for *PAI‐1* rs1799889 A/G polymorphism and VTE

					Genotype distribution (AA/AG/GG)			
First author, year	Country	Ethnicity	Type of disease	Sample size	Cases	Controls	*P*‐value for HWE	NOS score
Akar 2000	Turkey	Caucasian	DVT	136/113	38/77/21	28/57/28	0.925	7
Akhter 2010	India	West Asian	DVT	110/110	48/54/8	29/56/25	0.838	8
Alfirevic 2010	Croatia	Caucasian	VTE	100/105	31/52/17	33/55/17	0.457	7
Arslan 2011	Turkey	Caucasian	DVT	33/33	14/19/0	12/18/3	0.305	7
Barcellona 2013	Italy	Caucasian	VTE	402/466	72/265/65	61/305/100	<0.001	7
Bedencic 2008	Slovenia	Caucasian	VTE	295/223	93/157/45	63/115/45	0.569	7
Bezgin 2018	Turkey	Caucasian	VTE	310/287	74/160/76	81/137/69	0.460	7
Bezgin 2018	Turkey	Caucasian	DVT	247/287	59/130/58	81/137/69	0.460	7
Bezgin 2018	Turkey	Caucasian	PE	20/287	5/11/4	81/137/69	0.460	7
Chen 2005	China	East Asian	DVT	120/120	46/55/19	42/51/27	0.135	8
Cushman 2004	USA	Caucasian	VTE	138/469	37/68/33	119/248/102	0.202	9
D'Amico 2015	Italy	Caucasian	VTE	243/622	NA	NA	NA	7
Eroglu 2006	Turkey	Caucasian	DVT	45/80	10/30/5	33/41/6	0.161	7
Espinosa 2002	Spain	Caucasian	VTE	38/100	8/19/11	21/52/27	0.662	7
Farajzadeh 2014	Iran	West Asian	VTE	193/500	50/83/60	353/91/56	<0.001	8
Ferrara 2013	Italy	Caucasian	DVT	168/70	139/27/2	62/7/1	0.158	7
Folsom 2003	USA	Mixed	VTE	308/640	77/160/68	173/326/141	0.590	8
Giannaki 2013	Greece	Caucasian	VTE	102/102	NA	NA	NA	7
Grubic 1996	Slovenia	Caucasian	DVT	83/50	25/50/8	15/29/6	0.160	8
Gu 2014	China	East Asian	VTE	198/212	146/35/17	123/55/34	<0.001	8
Hasan 2006	Egypt	Caucasian	DVT	48/40	20/22/6	8/16/16	0.292	7
Kaya 2013	Turkey	Caucasian	VTE	80/79	19/43/18	29/36/14	0.628	7
Kotwal 2013	USA	Mixed	PE	12/12	NA	NA	NA	7
Kuhli‐Hattenbach 2017	Germany	Caucasian	VTE	25/241	6/16/3	56/122/63	0.837	7
Kumari 2014	India	West Asian	VTE	93/102	31/39/23	27/53/22	0.674	7
Kupeli 2011	Turkey	Caucasian	VTE	80/103	24/38/18	28/57/28	0.925	7
Kupeli 2011	Turkey	Caucasian	PE	51/103	15/27/9	28/57/28	0.925	7
Lichy 2007	Germany	Caucasian	VTE	76/195	21/40/15	61/91/43	0.413	7
Mansilha 2005	Portugal	Caucasian	DVT	81/88	22/40/19	21/39/28	0.311	7
Meglic 2003	Slovenia	Caucasian	VTE	30/53	7/16/7	14/29/10	0.464	8
Morange 2000	France	Caucasian	VTE	168/214	50/79/39	50/105/59	0.804	7
Oguzulgen 2009	Turkey	Caucasian	PE	143/181	36/63/44	46/94/41	0.595	7
Onur 2012	Turkey	Caucasian	VTE	28/50	12/10/6	17/19/14	0.093	8
Ozkan 2012	Turkey	Caucasian	VTE	158/134	91/56/11	66/57/11	0.789	8
Pop 2014	Romania	Caucasian	DVT	168/162	51/71/46	38/95/29	0.025	7
Prabhudesai 2017	India	West Asian	VTE	87/251	23/38/26	82/132/37	0.170	8
Ridker 1997	USA	Mixed	VTE	121/495	36/59/26	133/247/115	0.988	7
Ringelstein 2012	Germany	Caucasian	VTE	136/1054	44/72/20	326/521/207	0.964	8
Ringwald 2009	Germany	Caucasian	DVT	50/85	11/29/10	21/42/22	0.915	8
Russo 2015	Italy	Caucasian	VTE	113/101	26/68/19	31/51/19	0.807	7
Sartori 1998	Sweden	Caucasian	DVT	70/100	21/42/7	26/50/24	0.997	7
Sartori 2003	Italy	Caucasian	DVT	73/76	29/34/10	23/42/11	0.244	7
Schenk 2008	Germany	Caucasian	VTE	69/238	23/41/5	66/122/50	0.645	7
Seguí 2000	Spain	Caucasian	DVT	190/93	NA	NA	NA	7
Stegnar 1998	Slovenia	Caucasian	VTE	158/145	46/88/24	38/76/31	0.541	7
Tàssies 2000	Spain	Caucasian	VTE	59/100	17/29/13	27/52/21	0.662	7
Vesa 2016	Romania	Caucasian	DVT	127/114	42/51/34	26/66/22	0.089	8
Visanji 2000	UK	Caucasian	VTE	99/99	39/45/15	26/43/30	0.196	7
Visanji 2000	UK	Caucasian	PE	28/99	12/13/3	26/43/30	0.196	7
Vuckovic 2018	Serbia	Caucasian	VTE	100/100	NA	NA	NA	8
Yioti 2013	Greece	Caucasian	VTE	38/44	NA	NA	NA	7
Zhou 2005	China	East Asian	DVT	29/24	8/17/4	6/14/4	0.392	7

Abbreviations: DVT, deep vein thrombosis; HWE, Hardy‐Weinberg equilibrium; NA, not available; NOS, Newcastle‐Ottawa scale; *PAI‐1*, plasminogen activator inhibitor‐1; PE, pulmonary embolism; VTE, venous thromboembolism.

### 3.2 Meta‐analysis results

*PAI‐1* rs1799889 A/G polymorphism was found to be significantly associated with VTE in Caucasians (dominant comparison: AA vs AG + GG, odds ratio [OR] 1.20, 95% confidence interval [CI] 1.09‐1.32; recessive comparison: GG vs AA + AG, OR 0.84, 95% CI 0.76‐0.94; allele comparison: A vs G, OR 1.08, 95% CI 1.02‐1.15) and East Asians (dominant comparison: AA vs AG + GG, OR 1.60, 95% CI 1.17‐2.19; allele comparison: A vs G, OR 1.53, 95% CI 1.21‐1.93). Further analyses revealed similar significant associations in the DVT (recessive model: GG vs AA + AG, OR 0.73, 95% CI 0.53‐0.99; allele model: A vs G, OR 1.13, 95% CI 1.02‐1.25) subgroup, yet no any positive results regarding PE were detected in this meta‐analysis (see Table 2). We also performed stratified analyses to explore the effects of established risk factors of VTE on observed genetic associations between *PAI‐1* rs1799889 A/G polymorphism and VTE, and we found positive results in these with Factor V Leiden mutation, whereas no any significant associations were detected in these with cancer or these who recently had a major surgery operation.

**Table 2:** Results of overall and subgroup analyses for *PAI‐1* rs1799889 A/G polymorphism and VTE

		Dominant comparison (AA vs AG + GG)			Recessive comparison (GG vs AA + AG)			Over‐dominant comparison (AG vs AA + GG)			Allele comparison (A vs G)		
Population	Sample size	*P* value	OR (95%CI)	*I* ^2^ statistic	*P* value	OR (95%CI)	*I* ^2^ statistic	*P* value	OR (95%CI)	*I* ^2^ statistic	*P* value	OR (95%CI)	*I* ^2^ statistic
Overall	5731/9075	0.30	1.10 (0.92‐1.32)	78%	0.11	0.87 (0.73‐1.03)	65%	0.57	1.04 (0.92‐1.17)	55%	0.21	1.04 (0.98‐1.10)	55%
Caucasian	4460/6609	**0.0002**	**1.20 (1.09–1.32)**	49%	**0.003**	**0.84 (0.76‐0.94)**	35%	0.73	1.02 (0.93‐1.11)	21%	**0.01**	**1.08 (1.02‐1.15)**	31%
East Asian	347/356	**0.004**	**1.60 (1.17–2.19)**	34%	0.06	0.71 (0.50‐1.02)	21%	0.28	0.83 (0.60‐1.16)	38%	**0.0004**	**1.53 (1.21–1.93)**	49%
West Asian	483/963	0.66	0.75 (0.20‐2.80)	96%	0.54	1.36 (0.51‐3.65)	91%	0.80	1.12 (0.47‐2.66)	92%	0.56	0.75 (0.28‐1.99)	97%
DVT	1778/1645	0.06	1.16 (0.99‐1.36)	36%	**0.05**	**0.73 (0.53–0.99)**	55%	0.84	1.02 (0.88‐1.17)	46%	**0.02**	**1.13 (1.02–1.25)**	47%
PE	254/682	0.47	1.14 (0.80‐1.61)	0%	0.44	0.75 (0.36‐1.57)	65%	0.41	0.88 (0.64‐1.20)	0%	0.57	1.11 (0.77‐1.60)	55%
Factor V Leiden	993/1870	**0.01**	**1.29 (1.06‐1.57)**	5%	0.06	0.68 (0.46‐1.02)	65%	0.92	0.99 (0.83‐1.19)	0%	**0.03**	**1.24 (1.02‐1.52)**	60%
Cancer	264/297	0.91	1.03 (0.57‐1.87)	57%	0.46	0.81 (0.45‐1.43)	0%	0.91	0.98 (0.70‐1.38)	31%	0.46	1.10 (0.85‐1.42)	50%
Surgery	91/121	0.41	1.28 (0.71‐2.32)	0%	0.41	0.73 (0.35‐1.54)	0%	1.00	1.00 (0.52‐1.91)	0%	0.72	1.08 (0.71‐1.64)	0%

*Note*: The values in bold represent there is statistically significant differences between cases and controls.

Abbreviations: CI, confidence interval; DVT, deep vein thrombosis; NA, not available; OR, odds ratio; *PAI‐1*, plasminogen activator inhibitor‐1; PE, pulmonary embolism; VTE, venous thromboembolism.

### 3.3 Sensitivity analyses

We examined the stability of combined results by deleting one study each time and combining the results of the remaining studies. The trends of associations remained consistent in sensitivity analyses, which indicated that the combined results were statistically stable.

### 3.4 Publication biases

Funnels plots were employed to estimate whether our combined results may be influenced by publication biases. Funnel plots of every comparison were symmetrical, which indicated that the combined results were unlikely to be seriously impacted by overt publication biases (see Figure S1).

## 4 DISCUSSION

The A/G variant of rs1799889 polymorphism is associated with a guanosine insertion at the −675 site of the PAI.^15^ Past pre‐clinical studies also demonstrated that the transcriptional activity of A allele was significantly higher than that of the G allele.^16^, ^17^ So theoretically, it is possible that carriers of the A allele were more prone to thrombotic disorders compared to carriers of the G allele. Recently, many genetic association studies assessed association between *PAI‐1* rs1799889 A/G polymorphism and VTE, yet the findings were somehow conflicting. Thus, this meta‐analysis was performed by us to more comprehensively analyze relationship between rs1799889 polymorphism and VTE. The combined results demonstrated that *PAI‐1* rs1799889 A/G polymorphism was significantly associated with VTE in both Caucasians and East Asians. Further analyses obtained similar positive findings in these with DVT and these with Factor V Leiden mutation. The trends of associations remained consistent in sensitivity analyses, which indicated that the combined results were stable.

To better understand the combined results of this meta‐analysis, some points should be considered. Firstly, the etiology of VTE is complex, so we recommend future studies to conduct haplotype analyses and investigate gene‐gene interactions to more precisely analyze the effects of genetics on disease susceptibility.^18^ Secondly, environmental factors may also affect relationship between *PAI‐1* rs1799889 A/G polymorphism and PAI. Unfortunately, the majority of eligible publications only focused on genetic associations, so we could not estimate genetic‐environmental interactions in this meta‐analysis.^19^ Thirdly, this meta‐analysis was designed to assess associations between all *PAI‐1* polymorphisms and VTE. Nevertheless, only rs1799889 polymorphism was analyzed by us because no any other *PAI‐1* polymorphisms were studied by at least two different studies. Fourthly, it should be noted that in 2014, Wang et al also performed a meta‐analysis to investigate association between rs1799889 polymorphism and VTE.^20^ Since many related articles were published after this meta‐analysis, an updated study like ours is warranted. The sample size of the current meta‐analysis was around 50% larger than that of the previous meta‐analysis (14 806 subjects vs 9254 subjects), so our work should be considered as a valuable supplement to pre‐existing literatures. Consistent with findings of the previous meta‐analysis, we also confirmed that rs1799889 polymorphism was associated with an elevated susceptibility to VTE in both Caucasians and East Asians. The results of these two meta‐analyses indicated that *PAI‐1* rs1799889 polymorphism might serve as one of the predisposing factors of VTE. Subgroup analyses by established risk factors of VTE were also further performed by us. Nevertheless, since these analyses were only based on limited number of participants, the findings of these analyses should be considered as only exploratory, and further experimental studies should try to confirm these results. Besides, more stratified analyses should also be conducted by future meta‐analyses if there are sufficient data to support additional analyses for other established risk factors of VTE. Fifthly, although we also conducted subgroup analyses by type of disease in this meta‐analysis, it is noteworthy that studies only focused on PE were scare, so the results of subgroup analyses by type of disease should also be taken as exploratory. Future studies are still needed to confirm these findings.

Some limitations of this meta‐analysis should also be mentioned. Firstly, the results regarding associations between polymorphisms in *PAI‐1* rs1799889 polymorphism and VTE were based on combining unadjusted findings of eligible publications due to lack of raw data.^21^ Secondly, gray literatures were not searched. So although funnel plots of every comparison were symmetrical, it is still possible that the combined results may be affected by publication biases.^22^, ^23^

## 5 CONCLUSIONS

In summary, the combined results of this meta‐analysis proved that *PAI‐1* rs1799889 A/G polymorphism may serve as one of the predisposing factors of VTE in both Caucasians and East Asians, especially in these with DVT and these with Factor V Leiden mutation. Further studies with larger sample sizes still need to verify our findings. Besides, given that the pathogenesis of VTE is complex, despite our comprehensive analyses, we still recommend further studies to explore gene‐gene interactions and gene‐environmental interactions in the development of VTE.

## CONFLICT OF INTEREST

The authors declare no potential conflict of interests.

## AUTHOR CONTRIBUTIONS

Guangbin Huang and Xuejun Deng conceived of the study, participated in its design. Guangbin Huang and Pan Wang conducted the systematic literature review. Tao Li performed data analyses. Guangbin Huang and Xuejun Deng drafted the manuscript. All authors have read and approved the final manuscript.

## Supporting information


**Figure S1** Funnel plots.

## Data Availability

Data sharing is not applicable to this article as no new data were created or analyzed in this study.
