# Supplementary material for: Genetic association between plasminogen activator inhibitor‐1 rs1799889 polymorphism and venous thromboembolism: Evidence from a comprehensive meta‐analysis
Source: Clin Cardiol. 2019 Nov 8;42(12):1232–8. doi: 10.1002/clc.23282 (PMC6906978; doi:10.1002/clc.23282)
Supplement: Supplementary file 1 — Figure S1 Funnel plots. [file CLC-42-1232-s001.doc]

Funnel plots


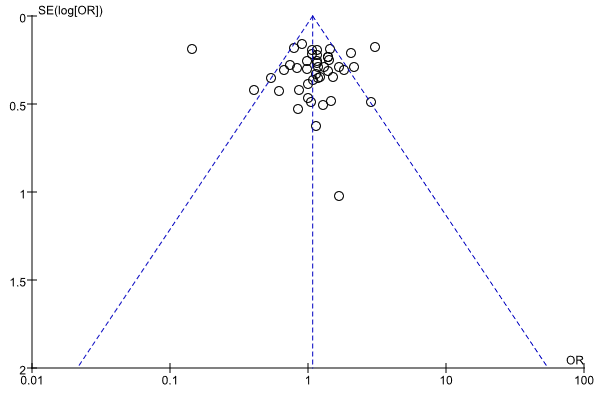


Funnel plot of **rs1799889** polymorphism and VTE under dominant comparison


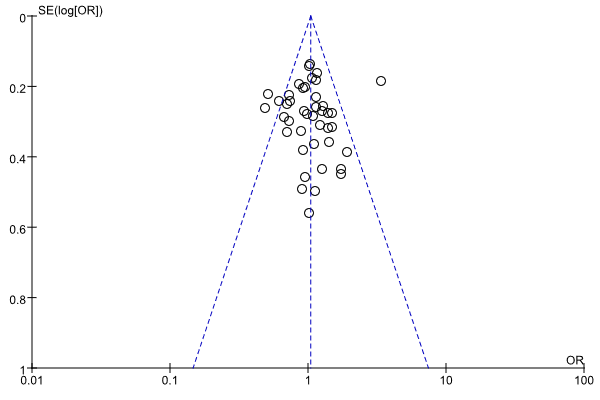


Funnel plot of **rs1799889** polymorphism and VTE under recessive comparison


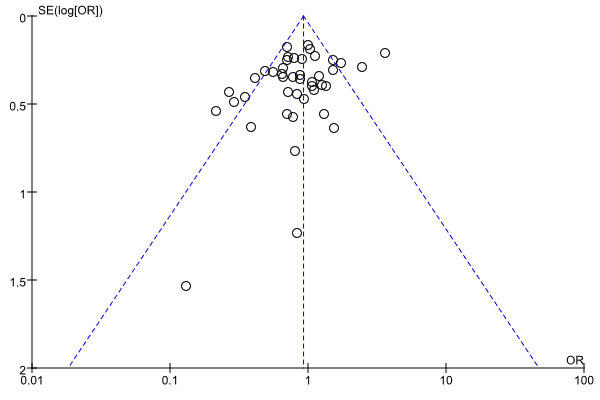


Funnel plot of **rs1799889** polymorphism and VTE under additive comparison


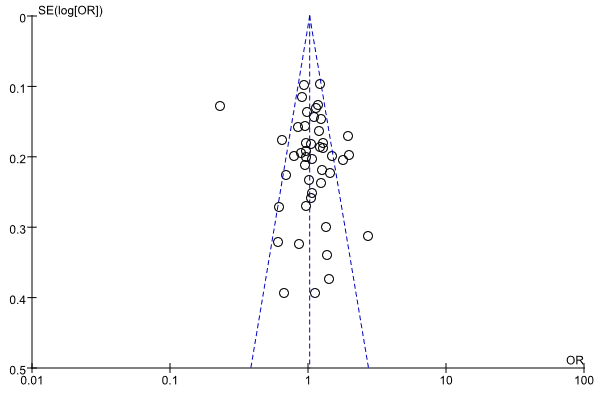


Funnel plot of **rs1799889** polymorphism and VTE under allele comparison
